# Development of the Modified Yale Food Addiction Scale Version 2.0 summary version in a representative sample of Czech population

**DOI:** 10.1186/s40337-020-00292-6

**Published:** 2020-05-04

**Authors:** Helena Pipová, Natália Kaščáková, Jana Fürstová, Peter Tavel

**Affiliations:** 1 Olomouc University Social Health Institute, Palacky University in Olomouc, Institut sociálního zdraví, Univerzitní 244/22, 771 11 Olomouc, Czech Republic; 2 Psychiatric Clinic, Bratislava, Pro mente sana Slovakia

**Keywords:** Food addiction, Yale food addiction scale, Czech population, Psychometrics

## Abstract

**Background:**

Food addiction receives attention because of its participation in the rising obesity prevalence that affects the quality of life. The Czech Republic’s prevalence of obesity belongs to the highest in Europe.

**Methods:**

We used the nationally representative non-clinical sample of 1841 respondents (*N* = 1841; 48,8% of men and 51.2% of women). Participants filled the sociodemographic questionnaire, Czech version of mYFAS 2.0 and the Relationship Questionnaire (RQ) to test the hypothesis.

**Results:**

Confirmatory Factor Analysis showed that the single-factor model of Czech version of mYFAS 2.0 had adequate fit indices (χ^2^ (55) = 9670.8, *p* <  0.001; CFI, TLI > 0.95; SRMR < 0.07; and RMSEA < 0.08). The sample reliability in our research corresponded to Cronbach’s α = 0.89 (95% CI 0.88–0.90). The youngest population (aged 15–19) had a significantly higher score of mYFAS 2.0, than the older age groups. People living alone scored significantly higher than the married respondents. The middle-income groups scored significantly lower in mYFAS 2.0 than the lowest income group. Occasional (social) smokers showed a higher mYFAS 2.0 score in comparison with non-smokers. A difference regarding attachment styles has also been revealed, respondents characterized by insecure attachment styles showed a higher YFAS score.

**Conclusion:**

The findings reinforce future work on the Czech version of mYFAS 2.0, including validation and using mYFAS 2.0 to explore food addiction and its related variables and attachment styles in the Czech environment.

## Plain English summary

This study is focused on food addiction and on its connection to attachment styles. The sample was nationally representative (1841 Czech respondents). The research revealed the mistake in the translation of one item of mYFAS 2.0, which is discussed in the article.

The results showed that people living alone scored significantly higher in food addiction than married respondents. The middle-income groups scored significantly lower in mYFAS 2.0 than the lowest income group. Respondents characterized by insecure attachment styles showed a higher YFAS score.

This study is the first one of this topic in the Czech national background.

## Background

Food addiction describes a psychological and behavioral eating pattern that is similar to abusing drugs [1–3]. The possible existence of food addiction is supported not only by clinical experience but also by animal testing and experiments involving human participants [4] and through research using imaging methods [5]. In 2014, 43.9% of adult men and 30% of adult women were diagnosed as overweight, and 19.3% of adult men and 18.2% of adult women were diagnosed as obese [6]. With a dramatic change in lifestyles and the unique development of technologies, the problems people are facing have changed rapidly. With a radical change in the food consumption environment [7] the problems may recently include problematic food consumption. Unlike many western countries, the Czech Republic has not paid attention to the issue, and so far there have not been such reliable methods to explore food addiction and attachment styles.

Although overweight and obesity represent the most apparent effects of food addiction, only some obese respondents show signs of food addiction or craving for food [5]. Burmeister et al. [8] showed that respondents with a higher score in food addiction tend to show significantly more severe problems with losing weight. Fairburn and Bohn [9] also revealed that less than 50% of the research participants diagnosed with food intake disorder (anorexia neurosa, bulimia, binge eating) comply with the criteria of food addiction. It shows that the concepts are intertwined, but do not overlap.

Ziauddeen and Fletcher [10] emphasize that some experts disagree with the concept of food addiction, since food does not show the psychoactive potential comparable to drugs, and moreover is less toxic than drugs. The term food addiction does not a priori define which food we should connect to the addiction-like behavior, however, the researchers assume that food rich in added fat and refined carbohydrates (sugar or white flour) may cause addiction-like behavior in more vulnerable individuals [11, 12].

The recent development related to food addiction, including the development and validation of an updated measure, mYFAS 2.0 has increased the awareness of the prevalence and association correlates of food addiction. YFAS 2.0 measures addiction-like eating based on new DSM 5 [13] where 11 diagnostic criteria for substance use disorder were listed. Schulte and Gearhardt [14] developed mYFAS 2.0 from YFAS 2.0 and claim that both versions show similar reliability levels and convergent validity. The Yale Food Addiction Scales are the only existing tools used for assessment of food addiction. Validation studies of modified mYFAS 2.0 were conducted in Brazil [15], in Spain [16], in Italy [17, 18] or in the USA [19].

The research on pathologic relation to food in the sense of addiction is of crucial importance because food as a substance necessary to sustain people is used in a way the effect of which proves to be contrary to life sustainability [7]. For many people, food has become recently a part of their emotional shield. It helps to cope with stress, ease fears and anxiety and provides a tranquil escape from everyday problems [20]. Research reveals that stress, anxiety and depressive mood show high comorbidity with addiction-like behavior related to food [21].

Eggert, Levendovsky & Klump [22] state that unhealthy coping mechanisms as overeating are associated with insecure attachment. It seems that people with insecure attachment styles have higher tendency to find the calm and “love” in food. Based on this theory we decided to measure also attachment in this study. A relationship between eating disorders and insecure attachment styles has been found [23] as well as an association between addictions and insecure attachment [24].

Bowlby’s theory [25] emphasized the fundamental role of the attachment system in the regulation of emotions and the establishment of self-esteem. Security in attachment develops from early relationships with nurturing adults who are sensitive and responsive to signals of distress. With inconsistent, non-responsive caregiving or in cases of child maltreatment, the insecure attachment can develop.

Although the individual differences in attachment dimensions of anxiety and avoidance are better described by dimensional than categorical models, the categorical approach is still often used, especially in clinical practice [26]. Bartholomew and Horowitz [27] described this four-category model of attachment styles, measured using the Relationships questionnaire. The categories of attachment styles represent different combinations of extreme positions of attachment anxiety and avoidance. A *secure* attachment style shows a relatively low score at both dimensions. *A preoccupied* attachment style shows a high score of attachment anxiety and a low score of avoidance. And the *dismissive* attachment style is characterized by a high score of avoidance and a low score of anxiety. Individuals experiencing a *fearful* attachment style show a combination of high anxiety and avoidance.

Insecurely attached individuals with higher attachment anxiety and/or avoidance can have less adaptive attachment-based strategies in relations to others and various non-adaptive behaviours including overeating can take on the function of regulating affect, as suggested by Maunder and Hunter [28].

## Methods

### The aim of the study

The aim of the current research was to create a Czech version of the modified Yale Food Addiction Scale 2.0 and test its psychometric properties in a nonclinical sample of the Czech population. Furthermore, the research explored the connection between food addiction, attachment style, and sociodemographic characteristics. Currently, there is no psychometrically sound tool for assessing food addiction in the Czech Republic.

### Participants

The sample (*n* = 1841 participants) was obtained from the population of the Czech Republic aged 15 or older. The participants were contacted based on random quota sampling. The sample is representative regarding age, sex, and regional affiliation. Genderwise, the sample comprised of 898 (48.8%) men and 943 (51.2%) women. Relative frequency has revealed a deviation of gender distribution at the maximum value of 0,1%.

This measuring was part of larger study which was focused on the social and psychological determinants of Czech population. The participants were selected randomly from all 14 administrative regions of the Czech Republic. As for regional affiliation relative to the distribution of citizens in the population, was stated the maximum deviation value at 0.1%.

### Description of process, intervention, and comparison

In total, 2225 citizens were randomly chosen with the help of quota sampling, out of that number 384 (i.e., 17.3%) citizens refused to participate in the study. The data was collected by trained administrators who administrated the self-report questionnaires to participants. The data were collected from October through December 2016. The informed consent was gained by participants before the administration process and their participation was anonymous and voluntary. The study design was approved by the Ethics Committee of the Olomouc University Social Health Institute, Palacky University in Olomouc (No. 2016/3).

### Measurements

#### Modified Yale food addiction scale 2.0

The scale consists of 13 items evaluated on an 8point scale labeled from never to every day. Each of the items is related to individual addiction criteria as stated by DSM 5 [13, 29, 30] or to a clinical significance. The mYFAS 2.0 scale was developed as a single-factor scale with CFA parameters of fit CFI = 0.96, TLI = 0.95, RMSEA = 0.08 [14].

According to DSM 5 [13], slight addiction is represented by 2–4 symptoms, mild addiction is represented by 4 to 5 symptoms and 6 or more symptoms represent serious addiction. All cases of food addiction diagnosed also need to be accompanied by the presence of a clinically significant decrease in physical fitness caused by the relationship to food. (Two questions of the scale, i.e., questions 5 and 6 monitor the clinical significance.)

The Czech version mYFAS 2.0 was obtained by the translation procedure. It was first translated into Czech by four official Czech language translation and those versions were compared, then followed by the completion of every item into the Czech language by researchers in order to create a single version of the tool. However, the comparative analysis revealed a translation mistake regarding the English term *distress* used within item number 5 ("My eating behavior caused me a lot of distress). Our translation of distress shifted the meaning close to *anxiety*, which has more narrow meaning than distress.

For this reason, some people might not find themselves in anxiety, but they might find themselves in distress. As a result, the question aiming at clinical significance could not be used in the current research. Since we could not use item number 5, we also did not use item number 6 (“I had significant problems in my life because of food and eating. These may have been problems with my daily routine, work, school, friends, family, or health.”) because they both have the same purpose in the questionnaire, to explore the clinical significance. For further research, the Czech translation is about to be modified. Nevertheless, items 5 and 6 are not necessary for psychometric assessment of the mYFAS 2.0 scale as they were not included in the original factor structure of mYFAS 2.0 [14].

#### Relationship questionnaire (RQ)

Szalai [31] conducted the review using the terms “eating disorder” and “attachment” from 1987 until 2017. From the 320 matches, he used 50 relevant studies which he integrated into his article. In our research, we focus on the topic of food addiction and since the concept of attachment was researched in connection with eating disorders, we were wondering about how the connection with food addiction is.

RQ is widely used in a variety of studies, and its Czech version was used within a wide-ranging study tracking transcultural differences in the distribution of individual relationship styles across 62 countries including the Czech Republic and Slovakia [32].

The Czech version of Re*lationships Questionnaire (RQ)* is a short questionnaire describing four relationship styles in four brief paragraphs the relationship styles being: secure, dismissing, preoccupied and, fearful [27]. In the first step, the participants were asked to select the style that best describes them. In the following step, they were asked to indicate the level of compliance with each style using a 7degree Lickert scale. For the analytic purposes, we used the mean values of variables indicating various attachment styles.

#### Sociodemographic data

The background characteristics gender, age income and other sociodemographic characteristics were obtained by the questionnaire.

### Statistical analysis of data

The factorial structure of the Czech mYFAS 2.0 scale was assessed by means of Confirmatory Factor Analysis (CFA) using a polychoric correlation matrix [33]. CFA was performed using the lavaan library in the R software. Given that all items have ordinal scales, a Diagonally Weighted Least Squares method (DWLS) was used to estimate the parameters of the CFA. To assess the quality of the factor model the following indices have been estimated: χ2 (Minimum Function Chi-square), CFI (Comparative fit index) and TLI (Tucker-Lewis index), SRMR (Standardized Root Mean Residual), and RMSEA (The Root Mean Square Error of Approximation). An acceptable model fit was considered CFI, TLI > 0.95; SRMR < 0.07; and RMSEA < 0.08 [34]. As measures of reliability, Cronbach’s alpha coefficients were assessed. Regression analysis was used to compare Czech mYFAS 2.0 mean values in various sociodemographic groups and for different attachment styles according to the RQ questionnaire. All regression models were adjusted for gender and age. The significance level was considered at *p* <  0.05. All analyses were performed using R 3.5.0 software.

## Results

### Factor structure and reliability

Psychometric properties are performed on the same version of the mYFAS questionnaire as in the original article [14]. The items that we have excluded from further analyses were not included in factor analysis of the original version either. The excluded items would be used to determine the clinical significance of food addiction, which was not the aim of this study.

The Czech mYFAS 2.0 scale consists of eleven items (without the items for clinical significance). The item analysis (means, standard deviations, and correlations with the scale) is presented in Table 1. There are two conventional ways of reporting the correlation of individual items with the scale: the raw correlation of the item with the entire scale, not corrected for item overlap, and the correlation of the item with the scale composed of the remaining items (the item of interest being dropped). There are of course disadvantages to the methods: item overlap inflates the correlation coefficient, and the scale is different for each item when an item is dropped. Thus, the third alternative is used, the correlation coefficient that corrects for the item overlap by subtracting the item variance, but then replaces it with the best estimate of common variance [35]. All items of the Czech mYFAS 2.0 scale have a satisfactory level of correlation with the scale. The correlation coefficients range from 0.55 (item 1) to 0.77 (item 7).

**Table 1:** Descriptive characteristics of the mYFAS 2.0 scale items: means, standard deviations, correlations of the items with the scale, and Cronbach’s alpha if an item is deleted

mYFAS 2.0 items	Mean	SD	Correlation with the Scale	Alpha when item dropped
1. Substance taken in larger amount and for longer period than intended	0.90	1.33	0.55	0.89
2. Persistent desire or repeated unsuccessful attempts to quit	0.90	1.37	0.60	0.89
3. Much time/activity to obtain, use, recover	0.26	0.89	0.69	0.89
4. Important social, occupational or recreational activities given up or reduced	0.63	1.15	0.69	0.88
7. Use continues despite knowledge of adverse consequences	0.26	0.82	0.77	0.88
8. Tolerance	0.43	1.07	0.74	0.88
9. Characteristic withdrawal symptoms; substance taken to relieve withdrawal	0.59	1.16	0.64	0.89
10. Continued use despite social or interpersonal problems	0.47	1.08	0.70	0.88
11. Failure to fulfil major role obligations	0.81	1.38	0.63	0.89
12. Use in physically hazardous situations	0.29	0.87	0.71	0.88
13. Craving, or a strong desire or urge to use	0.34	0.98	0.74	0.88

Note: *SD* standard deviation, Correlation with the Scale = item whole correlation corrected for item overlap and scale reliability

**Fig. 1:**
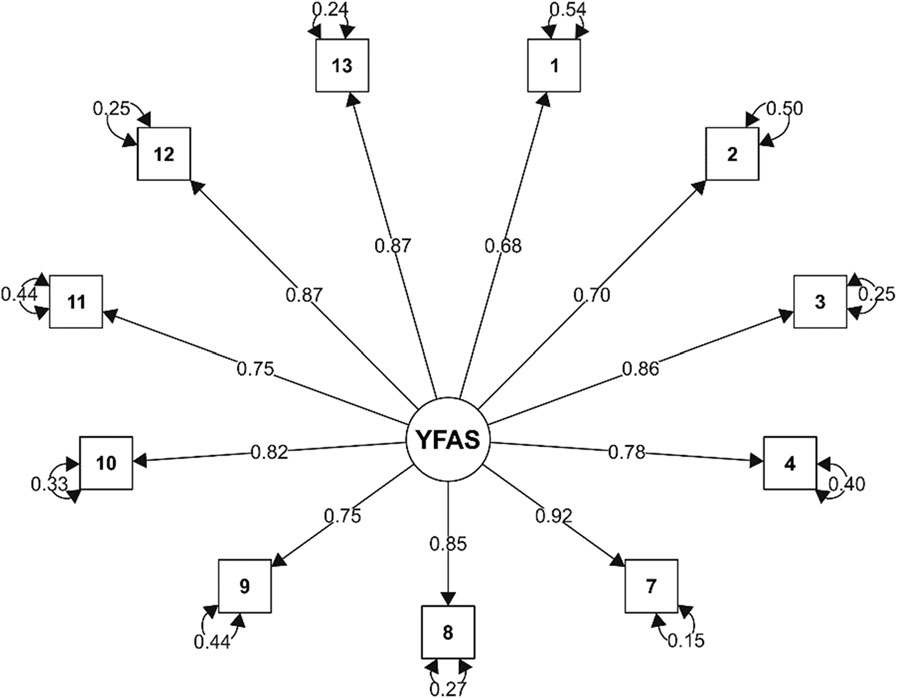
SEM path model of the Czech mYFAS 2.0 scale

The statistically significant result of Bartlett’s test of sphericity (χ^2^ (55) = 9670.8, *p* <  0.001) and the Kaiser-Meyer-Olkin Measure of Sampling Adequacy > 0.80 (KMO = 0.92) indicate that the data meet the conditions for using a factor analysis [33]. A unidimensional (one-factor) model was considered for the confirmatory factor analysis (CFA) according to Shulte and Gearhardt [14]. The CFA was performed on the polychoric correlation matrix, a Diagonally Weighted Least Squares method (DWLS) was used to estimate the parameters. In this CFA model, loadings of all items are medium to high (with values of 0.68–0.92). The model has demonstrated a satisfactory fit: CFI = 0.992, TLI = 0.990, SRMR = 0.058, RMSEA = 0.072 (90% CI 0.067–0.078). The SEM path model with one factor is presented in Fig. 1.

The internal consistency of the Czech mYFAS 2.0 questionnaire was verified with the Cronbach’s alpha coefficient. This analysis showed the high reliability of the scale in the Czech environment, with the Cronbach’s α = 0.89 (95% CI 0.88–0.90). The Cronbach’s alpha coefficients when individual items were deleted from the scale are presented in Table 1.

### Socio-demographic differences

Demographic characteristics of the data are presented in Table 2. The effect of different socio-demographic groups on the mean score of the Czech mYFAS 2.0 scale was assessed by linear regression. The socio-demographic groups were considered to be categorical predictors (with one category being the reference group) and the mean Czech mYFAS 2.0 score representing the continuous outcome variable. The attachment styles according to the RQ questionnaire were considered continuous predictors in the linear regression models. Thus, the standardized beta coefficients are referred to. All regression models (except for the gender and age models) were adjusted for gender and age. The results of the comparison are presented in Table 2.

**Table 2:** Descriptive characteristics of the data set and results of linear regression models of the mean Czech mYFAS 2.0 score in different socio-demographic groups (adjusted for gender and age)

	n (%)	mYFAS 2.0 Score Mean (SD)	Beta Coefficient	Std. Error	***p***-value
Total	1841 (100)	0.53 (0.79)			
**Gender**					
1. female	943 (48.8)	0.55 (0.86)	reference		
2. male	898 (51.2)	0.51 (0.72)	0.049	0.036	0.176
**Age**					
1. 15–19	100 (5.4)	0.71 (1.03)	reference		
2. 20–29	296 (16.1)	0.57 (0.84)	−0.145	0.090	0.108
3. 30–39	282 (15.3)	0.52 (0.76)	**−0.182**	0.091	**0.045**
4. 40–49	364 (19.8)	0.49 (0.76)	**−0.215**	0.088	**0.015**
5. 50–59	280 (15.2)	0.52 (0.75)	**−0.184**	0.091	**0.043**
6. 60–69	313 (17.0)	0.52 (0.77)	**−0.184**	0.089	**0.040**
7. 70 plus	206 (11.2)	0.45 (0.74)	**−0.249**	0.095	**0.009**
**Living Arrangement**					
1. in marriage	869 (47.2)	0.48 (0.74)	reference		
2. with partner	323 (17.5)	0.54 (0.72)	0.049	0.052	0.347
3. alone	334 (18.1)	0.59 (0.88)	**0.119**	0.050	**0.017**
4. with parents/siblings/family	315 (17.1)	0.59 (0.87)	0.078	0.056	0.163
**Marital Status**					
1. single	478 (26.0)	0.60 (0.87)	reference		
2. married	894 (48.6)	0.48 (0.74)	−0.090	0.055	0.104
3. divorced	234 (12.7)	0.56 (0.80)	−0.009	0.069	0.901
4. widow/er	177 (9.6)	0.46 (0.69)	−0.086	0.089	0.333
5. unmarried mate	58 (3.2)	0.68 (0.98)	0.108	0.109	0.323
**Education**					
1. primary	155 (8.4)	0.61 (0.96)	reference		
2. skilled operative	572 (31.1)	0.51 (0.79)	−0.100	0.071	0.156
3. high school	750 (40.7)	0.53 (0.79)	−0.107	0.069	0.119
4. college/university	364 (19.8)	0.51 (0.72)	−0.125	0.075	0.095
**Family Income (monthly)**					
1. < 10,000 CZK	78 (4.2)	0.72 (1.02)	reference		
2. 10,000–20,000 CZK	411 (22.3)	0.59 (0.84)	−0.138	0.096	0.151
3. 20,000–30,000 CZK	546 (29.7)	0.53 (0.85)	**−0.228**	0.095	**0.017**
4. 30,000–40,000 CZK	369 (20.0)	0.48 (0.64)	**−0.298**	0.099	**0.003**
5. 40,000–50,000 CZK	261 (14.2)	0.46 (0.69)	**−0.311**	0.102	**0.002**
6. 50,000–60,000 CZK	109 (5.9)	0.42 (0.70)	**−0.382**	0.117	**0.001**
7. 60,000–70,000 CZK	39 (2.1)	0.58 (0.87)	−0.212	0.154	0.167
8. > 70,000 CZK	28 (1.5)	0.62 (0.89)	−0.233	0.172	0.177
**Smoking**					
1. No	1315 (71.4)	0.51 (0.75)	reference		
2. Yes, every day	361 (19.6)	0.54 (0.86)	0.028	0.047	0.544
3. Yes, less than once a day	165 (9.0)	0.66 (0.92)	**0.131**	0.065	**0.044**
**RQ Attachment Style**					
Secure			−0.014^a^	0.013	0.559
Fearful			**0.161**^**a**^	0.011	**< 0.001**
Preoccupied			**0.175**^**a**^	0.011	**< 0.001**
Dismissive			**0.129**^**a**^	0.010	**< 0.001**

^a^Standardized coefficient

There were more men than women (51.2% vs. 48.8%) in the sample. There was no significant difference in the mean Czech mYFAS 2.0 scores between men and women. The highest mean score in Czech mYFAS 2.0 was obtained from the youngest group of participants. All age groups above 30 years had significantly lower mean scores than the reference group 15–19 years. The results in Table 2 show that there were no statistically significant differences in the marital status groups and the education groups either. In living arrangement groups, the lowest mean score was obtained from the respondents living in a marriage. People living alone had a significantly higher Czech mYFAS 2.0 score than the married. In different income groups, the mean Czech mYFAS 2.0 scores lowered with increasing income, but only to the point of 60,000 CZK monthly (approximately 3000 USD). Respondents with monthly income above 60,000 CZK did not have significantly lower mean of the Czech mYFAS 2.0 score than respondents in the lowest income group (< 10,000 CZK per month, approximately 500 USD). Smokers who smoke less than once a day scored significantly higher than non-smokers. On the contrary, regular everyday smokers mean Czech mYFAS 2.0 score was not significantly different from the non-smokers.

There was no association found between the secure attachment style and the mean Czech mYFAS 2.0 score. For the fearful, preoccupied and dismissive attachment styles, higher values in RQ questionnaire were associated with higher values in Czech mYFAS 2.0. With every increase of one SD in the fearful, preoccupied, or dismissive RQ items, the Czech mYFAS 2.0 mean score rises by 0.16 SD, 0.18 SD, or 0.13 SD, respectively.

## Discussion

This study aimed to translate, test, and assess the psychometric properties of the modified version of the Yale Food Addiction Scale 2.0 in the Czech environment and assess the relationship between food addiction and attachment styles.

In the current study, reliability analysis has shown high internal consistency. The overall reliability of the mYFAS 2.0 scale is similar to the one reported by Schulte and Gearhardt in their validation study [14]. Other studies show a similar level of internal consistency of mYFAS 2.0, including the Brazilian [15] and Italian [18]. Spanish [16] and the Italian [17] studies found high internal consistency also in YFAS 2.0.

Confirmatory factor analysis has supported a one-factor model of the scale. Both the Italian [18] and the Brazilian [15] versions of mYFAS 2.0 confirm the single structural model as well.

Due to the erroneous translation of question number 5, the respondents may have proved a higher tendency to answer the question negatively which would significantly lower the probability for them to be classified as addicted to food. The next study will be conducted with a different translation of distress.

### Gender

Our results show no differences in mYFAS 2.0 scores in gender, similarly to German studies of Schulte and Gearhardt [14], Carr et al. [36] and Hauck et al. [37]. These observations are contradictory to the conclusions of Gearhardt et al. [38], Nunes-Neto [39], Carr [36] and Pursey [40] who found a significant relationship for gender, with women reporting greater symptoms and diagnosis threshold scores of food addiction. The interesting finding that Czech women have a slightly lower score than the finding from for example Nunes-Neto [39], who also showed that Czech men are more probable to have FA than men other countries. Those results show cultural similarities and the significant influence of culture, which is similar to a German national, those are both central Europe countries.

### Age

Our results have shown the highest scores of Czech Modified Yale Food Addiction Scale 2.0 summary version in the youngest respondents (15–19 years). The research based on a representative sample of German population [37] found that people aged 18–29 years had the highest prevalence of food addiction measured by YFAS 2.0; the authors speculate that it might be caused by young people being influenced by modern food environment with well accessible high calories foods. Similar results were found in U.S. adults where food addiction had a higher prevalence in younger individuals [36].

Hoek and van Hoeken [41] mention that higher incidence of addiction-like behavior indicators for food may be related to the incidence of disordered eating, which shows a higher prevalence in respondents under 40 years of age. On the other hand, other studies [39, 42] have not found any differences in scores of food addiction in age groups.

### Living arrangement and marital status

People living alone have scored higher in the summary score of mYFAS in comparison to respondents living in a marriage. According to the findings of Dinçyurek, Alasya, and Kağan [43], when people are emotionally or socially lonely, they try to compensate these feelings by eating and seek comfort in food. Contradictory to these findings, Bereson et al. [42] did not find any difference between people living together and separated. Nunes-Neto [39] in his study assessing food addiction from many different views, did not find a difference in food addiction concerning marital status.

### Education

Regarding the level of education, our results have shown no differences in the food addiction score concerning the education level. This was confirmed in other studies as well [37–39].

### Family income

The highest scores of the mYFAS 2.0 in the Czech population were found in the lowest income groups. Bereson et al. [42] used YFAS to examine low-income, reproductive-aged women and found a lower prevalence of food addiction among low-income women compared to middle-income women. Schulte and Gearhardt [36] reported an association of higher food addiction prevalence with lower income white U.S. respondents. The higher score in Czech mYFAS 2.0 as shown in the lowest income population, may coincide with a stressful environment. Low family income poses a challenge to the basic psychological needs as defined by Maslow in [44]. This is especially true of the need for nutrition and security. Low income may urge respondents to economize on food, buying a low-quality, food rich in fat and refined sugars. Long-term consumption of junk food may make it more difficult for respondents to control their calorie intake because it gradually changes the satiety thresholds. Unlike those results, the study by Nunes-Neto [39] in the large sample did not show a difference in food addiction in different income groups.

### Smoking

Social smokers but not regular smokers have reported significantly higher mYFAS 2.0 score compared to non-smokers. Chao [45] found that current smokers reported a higher craving for palatable food and its intake. However, after adjusting for depression and stress, these findings were no more significant. Bereson et al. [42] did not find any differences of the score of food addiction in smokers and non-smokers in his sample of women, but he did not use the category of non- regular smokers. Regular cigarette smoking is generally associated with lower consumption of meals rich in sugar. On the other hand, based on empirical and also scientific knowledge, when regular smokers quit smoking, they increase their use of sugar and thus gain weight [46].

### Attachment style

We found a higher summary score of mYFAS 2.0 in the fearful, preoccupied and dismissive attachment styles. Elfang and Linné [47] explain that for the insecure attached respondents, food belongs to the ways of coping with stress and regulating their feelings.

Orzolek-Kronner [48] claims that people with insecure attachment style have more often disordered eating than those who have secure attachment. He explains that food brings comfort from pain and stressors and that emotional eating becomes a symptom of insecure attachment. According to the qualitative study conducted by Hernandez-Hons [49] insecurely attached people turn toward food instead of human.

## Conclusion

The results have shown the highest scores of Czech Modified Yale Food Addiction Scale 2.0 summary version in the youngest respondents (15–19 years) which are in unity with some of the research results, for example from Germany and the U.S. People living alone have scored higher in the summary the score of mYFAS in comparison to respondents living in a marriage, this might be because of compensation emotions and loneliness with food, not all researches confirmed these foundings. Regarding the sociodemographic such as education did not show any differences in food addiction.

The lowest income groups showed the highest scores of the mYFAS 2.0 in the Czech population which might be connected to economizing on food, buying a low-quality food, rich in fat and refined sugars This finding regarding an economy of the people connected to the way the eat can be an impulse for political changes in this field.

Regarding smoking variable, social occasional smokers show higher mYFAS 2.0 score compare to nonsmokers.

The results showed higher summary score of mYFAS 2.0 in the fearful, preoccupied and dismissive attachment styles. That might be because people with insecure attachment style have more often disordered eating than those who have a secure attachment. This result might become evidence based stimulus for the psychotherapy work.

These findings and also the mistake in the translation mistake reinforce future work on the Czech version of mYFAS 2.0, including validation and using mYFAS 2.0 in exploring food addiction and its related variables in the Czech environment.

### Strengths and limitations

This study has several significant strengths such as a large and representative sample and the fact that this is the very first study in the realm of food addiction which opens the door for further research in this field in the Czech environment.

The limitations consist mainly in a translation mistake of the item dedicated to the clinical significance, which resulted in the failure to validate the research results and subsequently it was impossible to state the prevalence levels. Another limitation of this study is the self-reporting method of data collection through the standardized controlled interview where respondents might be influenced by the social image they try to build.

The data collection for the researches we have cited in our study was organized as paper-based or computer-based. However, our research collected the data through standardized face to face interviews which could have significantly influenced the quality of the data collected. The respondents may have felt embarrassed at admitting their failure about food consumption. Moreover, the topic of problematic food consumption, especially excessive food consumption seen as the counterpart problem to anorexia or bulimia, is still seen as a taboo topic by many a Czech, even though many Czechs may face the problem themselves, which makes the self-evaluation very difficult limiting the possibility for many respondents to spot their problem and describe it accurately.

Pressmann et al. [50] add that the most significant threat to the validity of methods such as YFAS is their reliance to self-evaluation of the subjects since the respondents express their agreement or disagreement to the phenomenon of the abstinence symptoms which are often misunderstood by individual subjects. Similarly, Ziauddeen and Fletcher [10] state that the limitation of YFAS lies in the dichotomization of complex symptoms such as anxiety, nervousness or abstinence symptoms.

Despite doubting the validity of tools with dichotomous character, we can summarize that the Czech mYFAS 2.0 scale has proved to be a consistent single-factor tool showing adequate reliability levels, although we were only working with summary scores not with the complete tool, because of the translation error.

### Implications

In the following research, we are about to adjust the translation of the tool, proceed with its validation and subsequently state the prevalence of food addiction in the Czech population. Subsequent research steps include a choice of different sampling criteria resulting in gaining more detailed data (e.g., on a sample of clinical respondents).

For effective research in our environment, paper-based testing is recommended; however, it is also possible to collect the data online without the need for physical presence of the researcher. Next steps regarding the adaptation of YFAS2.0. or mYFAS2.0. include a study based in not only YFAS 2.0. but also, in other tools measuring the relationship to food and food behavior to be able to determine the discrimination validity of the tool.

## Data Availability

The datasets generated and/or analyzed during the current study are not publicly available but are available from the corresponding author on reasonable request.

## Abbreviations

mYFAS 2.0: The Modified Yale Food Addiction Scale Version 2
RQ: Relationship Questionnaire
